# Nuts as a Dietary Enrichment with Selected Minerals—Content Assessment Supported by Chemometric Analysis

**DOI:** 10.3390/foods11203152

**Published:** 2022-10-11

**Authors:** Renata Markiewicz-Żukowska, Anna Puścion-Jakubik, Monika Grabia, Jakub Perkowski, Patryk Nowakowski, Joanna Bielecka, Jolanta Soroczyńska, Grzegorz Kańgowski, Jakub M. Bołtryk, Katarzyna Socha

**Affiliations:** Department of Bromatology, Faculty of Pharmacy with the Division of Laboratory Medicine, Medical University of Białystok, Mickiewicza 2D Street, 15-222 Białystok, Poland

**Keywords:** nuts, calcium, magnesium, potassium, selenium, zinc, RVI, chemometric analysis, functional products, dietary supplementation, disease prevention

## Abstract

Nuts used as a snack and meal accompaniment supply plant protein and fatty acids that are beneficial for human health; however, they can also provide minerals. The aim of this study was to determine the content of selected elements that are often deficient in the diet (calcium, potassium, magnesium, selenium, and zinc) in nuts and determine whether they can be used to supplement deficiencies in the diet. In this study, we analyzed 10 types of nuts (*n* = 120 samples) that are consumed and available for sale in Poland. The content of calcium, magnesium, selenium, and zinc was determined by the atomic absorption spectrometry method, and flame atomic emission spectrometry was used for determination of potassium contents. The highest median calcium content was found in almonds (2825.8 mg/kg), the highest potassium content in pistachio nuts (15,730.5 mg/kg), the highest magnesium and selenium contents in Brazil nuts (10,509.2 mg/kg and 4348.7 μg/kg, respectively), and the highest zinc content in pine nuts (72.4 mg/kg). All the tested nuts are a source of magnesium, eight types of tested nuts are a source of potassium, six nut types are a source of zinc, and four nut types are a source of selenium; however, among the tested nuts, only almonds can be considered a source of calcium. Moreover, we found that selected chemometric methods can be useful in the classification of nuts. The studied nuts are valuable products that can be used to supplement the diet with selected minerals and can therefore be labelled as functional products crucial for disease prevention.

## 1. Introduction

Nuts is a general term for tree nuts or fruit seeds with an edible grain and an inedible hard shell [1]. Nuts are a widespread group of products consumed most frequently in the Mediterranean diet but recommended for populations worldwide [2]. The most common products of this group are almonds (*Amygdalus communis* L.), Brazil nuts (*Bertholletia excelsa* Humb. et Bonpl.), cashews (*Anacardium occidentale* L.), hazelnuts (*Corylus avellana* L.), macadamia nuts (*Macadamia* *ternifolia* F. v. Mueller), pecan nuts (*Carya illinoiensis* (Wangenh.) K. Koch), pine nuts (*Pinus pinea* L.), pistachio nuts (*Pistacia vera* L.), and walnuts (*Juglans regia* L.). Peanuts (*Arachis hypogea* L.) are botanically a legume but are identified by consumers as ‘nuts’ [3].

Nuts is a category of food products with a very wide range of uses, although most often associated with roasted or dried consumption, as an addition to confectionery (cookies, cakes, and chocolate), or hidden in commercial products (sauces, oils, ice creams, butter, and pastes). However, due to their nutritional value, they should be eaten as snacks and as a part of complete meals [4,5].

The first study that reported the beneficial effects of nut consumption on health was published almost 30 years ago [6]. It was a breakthrough moment because since then, research has been conducted on the intensive health effects of nuts. Investigation in the 1990s suggested that some civilizations consumed nuts even before cereal grains became part of their diet [5].

Some consumers are concerned about the consumption of nuts because they are fat-rich and therefore are a very energy-dense food. Although each fat provides the same amount of calories, they are not created equal. It has been scientifically proven that saturated and trans fatty acids have a negative effect on the body’s lipid metabolism. However, fats found in high concentrations in nuts, i.e., monounsaturated (MUFA) and polyunsaturated (PUFA) fatty acids, have beneficial effects on the health of consumers by improving the aforementioned metabolism—therefore, they have the opposite effect [7]. Nut consumption is often associated with increased health risks related to contaminants, such as aflatoxins produced by fungi; in the case of nuts, the most common are *Aspergillus flavus*, *A. parasiticus*, and *A. nomius* [8]. Based on reported cases in 2015–2020, the least contaminated nuts include Brazil nuts, pecans, and walnuts [8,9]. Nuts can also contain toxic elements. In our previous study, 8% of tested samples exceeded the lead limit, 33% of which were pecans. The remaining studied products were considered safe [10]. Nevertheless, there are many health benefits associated with adding nuts from verified sources to the diet. They contain dietary fiber, which has a beneficial effect on the intestines and their peristalsis, as well as a number of vitamins (E, K, B_1_, B_6_, and B_9_), minerals (e.g., calcium (Ca), potassium (K), magnesium (Mg), selenium (Se), and zinc (Zn)), and bioactive compounds (antioxidants, carotenoids, phytosterols, and phenols) [11].

In terms of energy value per 100 g of product, the most caloric nuts are macadamia and pecan (200 kcal). Taking into account nutritional density, the nuts with the highest content of PUFAs are walnuts (13 g); the highest MUFA content is found in macadamia nuts (17 g); the highest protein content is found in almonds and pistachio nuts (6.0 g and 5.8 g, respectively); the highest content of dietary fiber is found in almonds (4 g); the highest content of alpha-tocopherol is found in almonds (7.3 mg); the highest contents of vitamin B_6_ and B_9_ are found in pistachio nuts and hazelnuts (0.6 mg and 32 mg, respectively); and the highest contents of mineral elements, such as K and Fe, are found in pistachio nuts (291 mg) and cashews (1.9 mg), respectively [12].

Research databases consist mainly of works exploring individual nuts; however, there is a lack of comparative studies on the most common types of nuts, particularly in terms of minerals, mainly Se content. Moreover, there is little information available about the contents of mineral in nuts, such as pecans or pine nuts, which were analyzed in this study. To the best of our knowledge, no studies have been conducted to date on the parameters of %RVI and INQ, evaluating nuts as a source of minerals in the diet.

Therefore, the aim of our study was to determine the content of the most important minerals essential for the maintenance of human health (Ca, K, Mg, Zn, and Se) in the most commonly consumed types of nuts. We also evaluated nuts as a potential dietary source of these elements, which are very often deficient in the diet, and determined whether the tested nuts can be used as dietary supplementation. Additionally, we supported our results with chemometric analysis.

## 2. Materials and Methods

### 2.1. Materials—Collection and Preparation

The study included 120 samples of nuts: 12 samples of 10 types of nuts (almonds, Brazil nuts, cashews, hazelnuts, macadamia nuts, peanuts, pecans, pine nuts, pistachio nuts, and walnuts). Packed nuts were purchased in stationery stores in Poland. The country of origin was specified on 81 packages: Argentina (*n* = 1), Australia (*n* = 11), Azerbaijan (*n* = 1), Bolivia (*n* = 2), Brazil (*n* = 5), China (*n* = 10), Georgia (*n* = 5), India (*n* = 7), Iran (*n* = 8), Italy (*n* = 1), Pakistan (*n* = 1), Poland (*n* = 8), Spain (*n* = 1), Ukraine (*n* = 1), USA (*n* = 16), and Vietnam (*n* = 3).

The samples of nuts were ground in a mortar and weighed in quantities of approximately 0.3 g (with an accuracy 1 mg) into mineralization vessels; exact masses were noted. Then, 4 mL of spectrally ultrapure concentrated nitric acid (69% HNO_3_, Tracepur, Merck, Darmstadt, Germany) was added to the vessels, and the samples were subjected to a wet mineralization process using the microwave technique in a closed system (Speedwave, Berghof, Eningen, Germany). The mineralized samples were stored at −20 °C in polypropylene vessels until analysis.

### 2.2. Ca, K, Mg, Se, and Zn Determination

The content of minerals in the nuts was determined by the atomic absorption spectrometry (AAS) technique with Zeeman background correction (Z-2000, Hitachi, Tokyo, Japan) using the method of atomization in an acetylene-air flame (Ca, Mg, Zn) or graphite tube (Se). Ca, Mg, Se, and Zn contents were determined at a wavelength of 422.7 nm, 285.2 nm, 196 nm, and 213.9 nm, respectively. A lanthanum chloride heptahydrate (Merck, Darmstadt, Germany) solution in a concentration of 1% was used as the modifying reagent to determine Ca and Mg contents. In the case of Se, a palladium-magnesium matrix modifier was used: 900 mg/L magnesium nitrate hexahydrate (Sigma-Aldrich, Steinheim, Germany) and 1500 mg/L palladium (II) nitrate hydrate (Merck, Darmstadt, Germany). Flame atomic emission spectrometry (FAES) was used to determine K contents at a wavelength of 766.5 nm. 

Standard calibration curves were prepared using a 1 g/L standard solution of Ca, K, Mg, Se, and Zn (Merck, Darmstadt, Germany). The limit of detection for of Ca, Mg, Se, and Zn determination, estimated as characteristic concentration, was 0.149 mg/kg, 0.01 mg/kg, 1.6 µg/kg, and 0.016 mg/kg, respectively. The detection limit for K was 0.03 mg/kg. Ultrapure water from a Simplicity UV water purification system (Millipore, Molsheim, France) and spectral-grade reagents were used in all the experiments.

### 2.3. Verifying the Accuracy of the Method

The accuracy of the method was verified using Simulated Diet D certified reference material (Livsmedelsverket, National Food Agency, Sweden). The contents of individual elements in the reference material were determined, and the results were compared with the concentrations listed on the certificate. The average percent recoveries for analytical methods used in the examination of Ca, K, Mg, Se, and Zn contents were 98.5, 99.4, 102.5, 101.6, and 103%, respectively. The precision of methods for determination of Ca, K, Mg, Se, and Zn were 3.2, 2.8, 2.6, 2.6, and 3.7, respectively. The RSD (relative standard deviation) value did not exceed 5%.

### 2.4. Assessment of Nuts as a Source of Minerals in the Diet

In order to assess the use of nuts as natural food products for supplementation of minerals in the diet, the following parameters were calculated: percent of recommended dietary allowance (%RDA) [13], percent of reference value intake (%RVI) [14,15], and index of nutritional quality (INQ) [16].

RDA determines the level of consumption of a nutrient that covers the needs of almost all people in the group, depending on age and gender [13].

Food products can be considered a source of mineral components when the consumption of their standard portion covers more than 15% of RVI for minerals: Ca (800 mg), K (2000 mg), Mg (375 mg), Se (55 µg), and Zn (10 mg) [14,15]. We adopted a portion weight of 42 g as the most common amount used in dietary intervention studies [11].

The INQ is a ratio of the nutrient-to-calorie content of foods [16]. It can be calculated according to the following formula:

(1) $$\mathrm{INQ}=\frac{A\;\times \;B}{C\;\times \;D}$$

where:

A—content of the tested component in 100 g of the product based on the results obtained in this study;

B—the standard of energy demand depending on gender, physical activity, age, and body weight (for women: 2100 kcal; for men: 2600 kcal);

C—energy value provided by 100 g of the product (for almonds: 604 kcal; Brazil nuts: 656 kcal; cashews: 553 kcal; hazelnuts: 666 kcal; macadamia nuts: 718; peanuts: 610 kcal; pecans: 691 kcal; pine nuts: 691 kcal; pistachio nuts: 621 kcal; walnuts: 666 kcal);

D—the requirement for the tested component depending on age and gender (for women: 1000 mg Ca, 3500 mg K, 310 mg Mg, 55 µg Se, and 8 mg Zn; for men: 1000 mg Ca, 3500 mg K, 400 mg Mg, 55 µg Se, and 11 mg Zn).

An INQ value above 1 indicates that the product is a good source of nutrients and can be used to supplement dietary deficiencies.

### 2.5. Statistical Analysis

The obtained data were statistically analyzed using Statistica software (Tibco, Palo-Alto, CA, USA). The results are presented as average value (A.V.) with standard deviation (SD), minimum (Min.), maximum (Max.), median (Med.), and first and third quartiles (Q1–Q3). The normality of the distribution of the data was checked by the Shapiro–Wilk test. Kruskal–Wallis analysis of variance (ANOVA) with post hoc analysis was performed to demonstrate the differences in the mineral content between the types of nuts. The relationship between the content of the examined elements was assessed on the basis of Spearman’s rank correlation. Differences at a *p*-value < 0.05 were considered statistically significant.

The next step was chemometric analysis, including cluster analysis (CA), principal components analysis (PCA), and discriminant analysis (DA). Cluster analysis was performed on the basis of the single-bond agglomeration method, and the distance measure was the Euclidean distance.

## 3. Results

### 3.1. Ca, K, Mg, Se, and Zn Contents

Table 1 presents a comparison of the content of the tested elements in 10 types of nuts.

The median Ca content in the examined nuts ranged from 145.3 mg/kg in pine nuts to 2825.8 mg/kg for almonds. Brazil nuts, hazelnuts, pistachio nuts, and walnuts were characterized by Ca contents above 1000 mg/kg.

Among all tested nuts, pistachios are the most K-rich (15,730.5 mg/kg), whereas macadamias have the lowest K content (4591.7 mg/kg).

The lowest Mg content was found in pistachio nuts (1463.3 mg/kg), and the highest Mg content was found in Brazil nuts (10,509.2 mg/kg) and cashews (7281.3 mg/kg).

Brazil nuts also had the highest Se content (4348.7 μg/kg), which was about 12 times higher than that observed in cashews (353.9 μg/kg) and 121 times higher than that in almonds (36.8 μg/kg).

Among the examined nuts, the lowest Zn content was found in macadamia nuts (15.7 mg/kg) and the highest in pine nuts (72.4 mg/kg), with high Zn content also found in cashews (67.2 mg/kg) and Brazil nuts (61.6 mg/kg).

### 3.2. Nuts as a Source of Minerals in the Diet

In the case of Ca, the RDA was ranged from 0.6% (pine nuts) to 13.0% (almonds). Among all types of nuts tested, only almonds can be considered a source of Ca, as one portion met RVI at 16% (Table 2). The calculated INQ for men and women was above 1 in almonds only.

The consumption of a standard portion of nuts provides between 5.6% (macadamia nuts) and 18.8% of the RDA (pistachio nuts) for K. All the nuts, except pecans and macadamia nuts, can be considered a source of K (a serving covers more than 15% of RVI). Four types of nuts (pistachio nuts, almonds, cashews, and hazelnuts) can supplement K deficiencies in the diet for men and women, taking into account the INQ value of.

On the basis of the determined content of Mg in nuts, consumption of a standard portion provides 15.9% (pistachios, for men) to 159.9% of the RDA (Brazil nuts, for women). Due to the relatively high content of Mg, all the nuts analyzed in this study can provide more than 15% RVI. In addition, according the INQ values of the studied nuts, they can all be used to supplement Mg deficiencies in the diet.

A recommended 42 g daily portion of nuts fulfills the RDA for Se from 3.8% (hazelnuts) to 348.7% (Brazil nuts). Only four types of nuts can be considered a source of Se in the diet (Brazil nuts, cashews, macadamia nuts, and pistachio nuts), providing 22 to 349% RVI. The above-mentioned types of nuts, as well as peanuts, had an INQ above 1.

Taking into account the determined Zn content in the nuts, the RDA coverage was assessed, which ranged from 6.2% (macadamia nuts, for men) to 41.3% (pine nuts, for women). Most of the tested nuts (almonds, Brazil nuts, cashews, peanuts, pecans, and pine nuts) cover more than 15% of the RVI. All types of nuts, except for macadamia and pistachios, had an INQ above 1.

Brazil nuts have high contents of Se and Mg—to cover 100% of the RDA, it is enough to consume only 12 g and 26.3–33.9 g, respectively. However, in the case of Zn, a portion of 101.8 g to 140.0 g will cover 100% of the RDA (Table 3).

### 3.3. Chemometric Analysis

The first step of chemometric data analysis was to assess the correlation between the content of elements in the tested nuts. We observed a positive, significant relationship between the content of Mg and Zn in pine nuts and pistachio nuts, as well as between all the studied types. High Zn content also correlated with the high Ca content in Brazil nuts and pecans. Moreover, high Ca content was correlated with high K content in all tested samples (Table 4).

In order to classify the examined nuts in terms of the content of beneficial elements, as well as to identify relationships between these factors, a chemometric analysis was performed, including CA, PCA, and DA.

**Figure 1:**
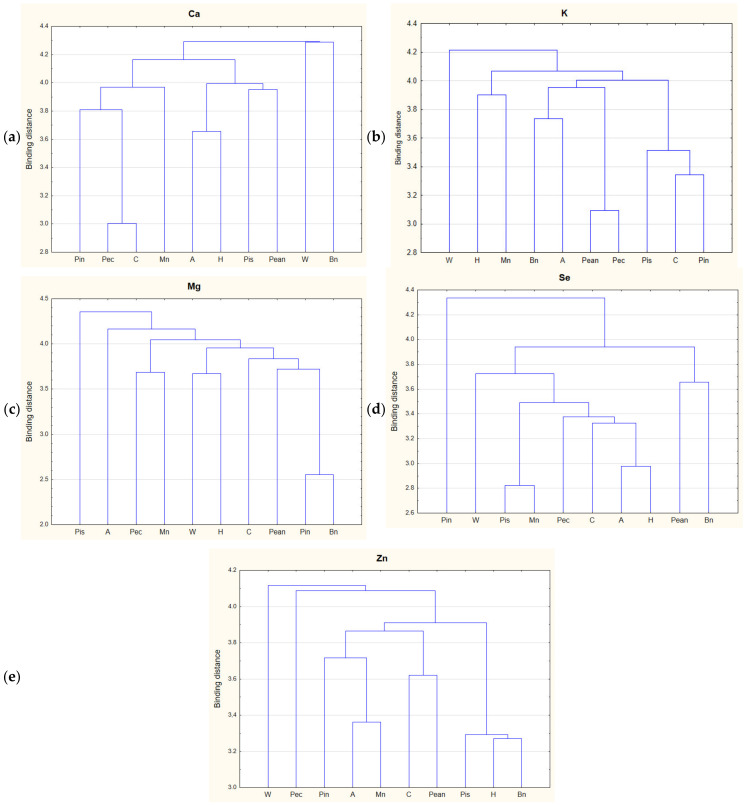
Analysis of the clusters of tested nuts: (**a**) calcium, (**b**) potassium, (**c**) magnesium, (**d**) selenium, and (**e**) zinc. Abbreviations: A—almonds, Bn—Brazil nuts, C—cashews, Ca—calcium, H—hazelnuts, K—potassium, Mg—magnesium, Mn—macadamia nuts, Pean—peanuts, Pec—pecans, Pin—pine nuts, Pis—pistachio nuts, Se—selenium, W—walnuts, Zn—zinc.

CA can distinguish objects into groups. The results of the chemometric analysis are presented in the form of a dendrogram in Figure 1. The criteria used allowed for the identification of characteristic clusters. In the case of the analysis of Ca contents in nuts, two main clusters were observed: one for Brazil nuts and walnuts (with medium Ca content). CA for Mg distinguished two clusters, including one with pistachios (the lowest average Mg content). In the case of Se, clusters were distinguished, including separate clusters for Brazil nuts (with the highest Se content) and for peanuts (with medium values). CA in terms of Zn content distinguished walnuts (with medium values) and pecans (with almost the highest maximum value). CA analysis on the basis of the potassium content distinguished a main cluster containing W.

**Figure 2:**
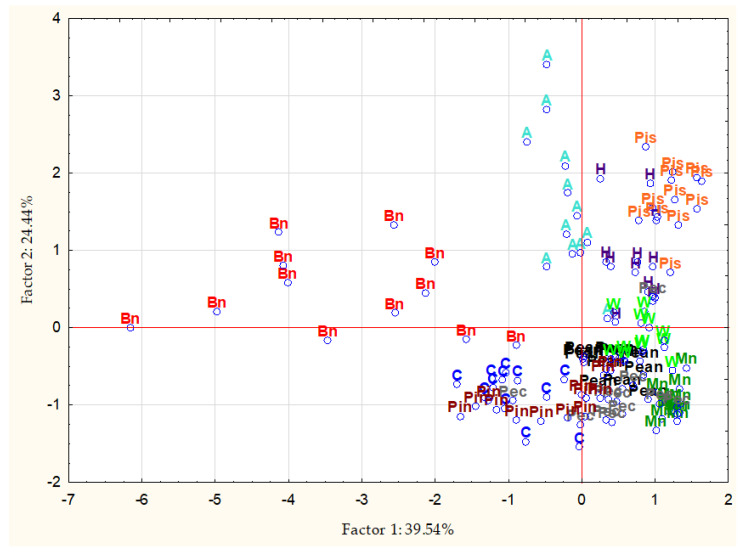
Principal component analysis scatter plot for factors 1 and 2. Abbreviations: A—almonds, Bn—Brazil nuts, C—cashews, H—hazelnuts, Mn—macadamia nuts, Pean—peanuts, Pec—pecans, Pin—pine nuts, Pis—pistachio nuts, W—walnuts.

The purpose of PCA is, inter alia, to reduce variables characterizing the studied cases. A high correlation was observed between the first three variables (Mg and Zn, r = 0.55; and Mg and Se, r = 0.63). The components corresponding to the first two factors explain 64.0% of the total variance, three factors explain 83.1% of the total variance, and four factors explain 95.0% of the total variance. In accordance with the Kaiser criterion, the components with eigenvalues greater than 1 were classified for interpretation; therefore, the quality of the representation was 77.3%. Figure 2 presents a plot of factor coordinates for the cases for factors 1 and 2. The Mg variable has a high value of the coefficient fifth (0.75), the variable Zn—the fourth (0.44), the variable Se—the third (0.56), the variable Ca—the second (0.74), and the variable K—the second (0.60).

The last analysis performed was the DA. In the case of examining the content of 5 elements in 10 types of nuts, the discrimination was highly significant (Wilks’ Lambda: 0.004, F = 29.995, *p* < 0.0001).

**Figure 3:**
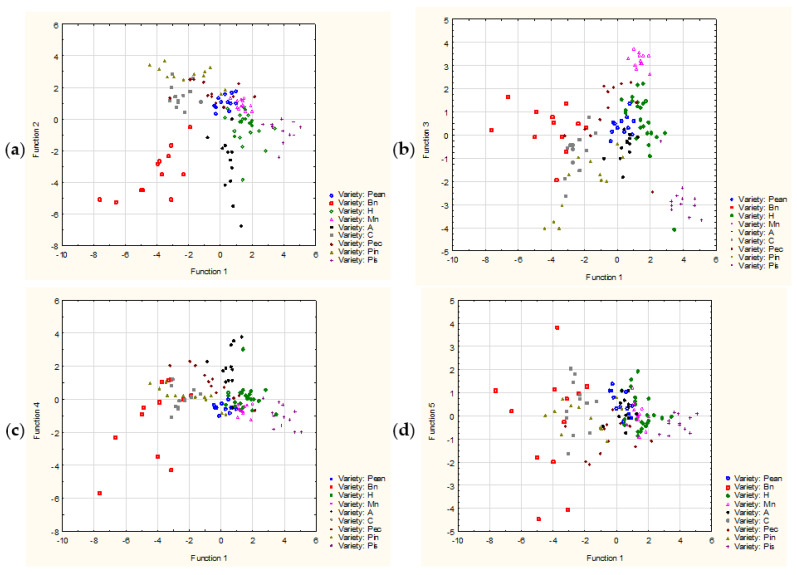
Discriminant analysis scatter plots for factors 1 and 2 (**a**), 1 and 3 (**b**), 1 and 4 (**c**), and 1 and 5 (**d**). Abbreviations: A—almonds, Bn—Brazil nuts, C—cashews, H—hazelnuts, Mn—macadamia nuts, Pean—peanuts, Pec—pecans, Pin—pine nuts, Pis—pistachio nuts, W—walnuts.

All variables were highly significant and were classified into the model. All discriminant functions were significant. The first function was responsible for 41% of the explained variance, two factors explained 71% of the total variance, three factors explained 92% of the total variance, and four factors explained 99% of the total variance. The mean values of the canonical variables enabled determination of which types of nuts were best distinguished by each of the functions: function 1 distinguishes mainly Brazil nuts, function 2 distinguishes Brazil nuts, function 3 distinguishes macadamia nuts, function 4 distinguishes Brazil nuts and pistachio nuts, and function 5 distinguishes pecans, as shown in Figure 3.

## 4. Discussion

The elements investigated this study are necessary for the proper growth and functioning of the human body. Unfortunately, their dietary intake is often insufficient, which is a global problem.

Janowska-Miasik [17] reported that Ca and Mg intake is below the recommended intake among the adult population of Poland. Additionally, according to our previous research, 28% of elderly subjects presented with Zn deficiency [18]. Globally, it is estimated that 17.3% of the population has inadequate Zn intake, with the highest proportions in Africa (23.9%) and Asia (19.4%). Pregnant females and their young children are the highest-risk groups for Zn deficiency [19]. Se deficiency affects between 500 million and 1 billion people worldwide due to inadequate dietary intake. The average intake of Se in Eastern Europe is lower than that in Western Europe [20,21]. Therefore, supplementing the diet with these ingredients seems to be necessary. However, nutrients from supplements may exhibit different physiological responses and absorption compared to nutrients found in food [19].

Currently, natural sources of nutritional ingredients are highly valued and recommended. Our research confirms the viability of nuts as a source of bioavailable minerals.

Most authors express the content of elements as an average content; therefore, in the following discussion, we refer to the average content values obtained in our study. Table 5 shows a comparison of our results with literature values.

Almonds are distinguished from other nuts with the highest average Ca content (3099.1 ± 1210.4 mg/kg). Other authors reported mean values ranging from 1491 to 3450 mg/kg. In the case of Mg, the values reported by most authors were twofold lower (2360–2650 mg/kg) than those obtained in the present study (5424 mg/kg), Moodley et al. [22], who reported similar values. The Zn contents obtained in the present study were similar to those reported by other researchers. Based on our study results, it can be concluded that the investigated nuts are a source of Ca, K, Mg, and Zn and can be labelled as functional products that can be used to effectively supplement the diet with these elements. Studies have shown, among other things, that due to the content of these elements, almonds have a significant effect in terms of improving not only lipid metabolism but also carbohydrate-insulin metabolism [23,24,25,26,27,28,29]. In patients with type 2 diabetes mellitus (T2DM), consumption of these nuts in the form of a snack (43 g serving) reduced postprandial blood glucose levels and increased the feeling of fullness without weight gain [27]. In patients treated with statins, the daily consumption of 100 g of almonds for four weeks did not affect body weight but improved high-density lipoprotein (HDL-c) levels and, by improving the parameters of lipoproteins, reduced the risk of cardiovascular diseases (CVD) [30]. Li et al. conducted a study including people with T2DM and reported that consumption of 60 g of almonds per day for four weeks reduced serum insulin concentration by 4.1% and the HOMA-IR index of insulin resistance by 9.2% compared to the control group. Moreover, the authors reported a reduction in total cholesterol (TC) and low-density lipoprotein (LDL-c) [26]. In people with coronary artery disease, consumption of 10 g per day for 12 weeks increased HDL-c and decreased TC, LDL-c, and triglycerides (TG) [31].

In Brazil nuts, other authors significantly lower contents of all analyzed elements than those reported in the present study. Furthermore, these nuts were identified by us as an excellent source of Se, K, Mg, and Zn in the diet. The addition of one item to the diet may have a positive effect on increasing the level of Se and the activity of glutathione peroxidase (GPx) [32,33]. This was observed in obese women and was not affected by the genotype associated with the Pro198Leu polymorphism [32]. Huguenin et al. reported a decrease in oxidative stress, the level of oxidized-LDL, and blood pressure (BP) and an increase in the activity of GPx in the blood in patients with hypertension and dyslipidemia who consumed 13 g per day for 12 weeks [33].

However, it should be emphasized that Se is an element with a narrow safety margin. Consumption of approximately 12 g of Brazil nuts (about 3 pieces) is sufficient to meet 100% of RDA. Therefore, long-term consumption of large quantities of these nuts, which are characterized by a high Se content, may result in a health-hazardous increase in serum Se levels [34].

In our study, cashews were characterized by a higher content of Zn, K, and Mg compared to the values reported in studies by other authors. These nuts had a higher content of Zn and Mg compared to data obtained by other authors and may be a rich source of these elements. Consumption of cashews for 28 days corresponding to 11% of the daily energy requirement (28–64 g/day) in patients with mild hypercholesterolemic disorders improved of lipid metabolism, i.e., TC and LDL-c were reduced [35]. A meta-analysis of three studies by Jalali et al. showed that the consumption of 30–42 g per day significantly reduced systolic BP but did not affect the lipid metabolism or diastolic BP in any of the studied groups (healthy adults and people with metabolic syndrome (MetS) and T2DM) [36].

Hazelnuts can be successfully used for the prevention of Mg deficiency. The contents of Mg and K reported in the present study were 2-fold and 1.5-fold higher than the values reported by other authors, respectively. In a 12-week study by Tey et al., no significant effect of hazelnut consumption on inflammatory markers and cell adhesion molecules was reported in overweight and obese people, regardless of the dose (30 and 60 g/day). However, their consumption improved the quality of the diet, although no adverse changes in body composition were observed. Consumption of 30 g per day of nuts is possible without losing the ‘desire to consume’ and ‘overall liking’ compared to a dose of 60 g/day [37].

In addition to Brazil nuts and cashews, macadamia and pistachio nuts proved to be a good source of Se. Garg et al. showed that monthly consumption of macadamia nuts in the amount of 15% of the daily energy requirement (40–90 g/day) in men with hypercholesterolemia favorably changed the biomarkers of oxidative stress, thrombosis, and inflammation, as well as risk factors for coronary heart disease, but also resulted in a reduction in TC and LDL-c and an increase in HDL-c [38,39].

Peanuts, as well as hazelnuts, can also be classified as functional products with the capacity to supplement the diet with Mg and K. Introducing peanuts, especially with high olein content, into a 4-week low-energy diet improved anthropometric parameters (body mass index (BMI), waist-to-hip ratio, and body fat tissue) of overweight adult men [40]. High-olein peanuts, in particular, increased diet-induced thermogenesis in overweight and obese men, purportedly by increasing the expression of disjoint protein (UCP) genes, in addition to reducing hunger through energy intake and increasing satiety [41]. Reis et al. conducted a study that investigated the glycemic response to peanut consumption in young people with normal BMI. Consumption of 63 g/day led to a reduction in carbohydrate consumption and postprandial glycemia [42]. Including them in the diet will not only improve the quality of the diet but also the feeling of fullness and increase thermogenesis [40,43,44].

Despite obtaining lower values in terms of Mg and Zn content in pecans compared to literature data, they have been shown to be a source of these components. Morgan and Clayshulte conducted a study on persons without lipid disorders. Subjects were randomly assigned to a test group that consumed 68 g of pecans per day for 8 weeks or to a control group that did not eat any nuts. In the pecan group, after 8 weeks, a decrease in LDL-c was demonstrated (from 2.61 ± 0.5 to 2.46 ± 0.6 mmol/L; *p* < 0.05). Moreover, the levels of TC and HDL-c in the study group was significantly lower than those in the control group (4.22 ± 0.8 vs. 5.02 ± 0.5 mmol/L; 1.37 ± 0.2 vs. 1.47 ± 0.3 mmol/L, *p* < 0.05), respectively) [45]. Rajaram et al. proved that, inter alia, introducing pecan nuts (72 g) to the diet for 4 weeks reduced TC and LDL-c by 0.32 mmol/L and TG by 0.14 mmol/L, in addition to increasing the concentration of HDL-c by 0.06 mmol/L [46].

The novelty of our study is the inclusion of pine nuts in the analysis, which have been investigated in only a few studies to date. Our research revealed that they can be a good source of Mg, K, and Zn. Lee et al. tested the effect of a dietary enrichment of 30 g/day of a mixture of nuts (walnuts, peanuts, and pine nuts) on metabolic markers in people with MetS and BMI of at least 23 kg/m^2^ for 6 weeks. Among women, TC and nonHDL-c levels improved in the nut group compared to the control group not consuming this product [47]. Using the semi-quantitative food frequency questionnaire, Jung et al. assessed the total consumption of nuts (peanuts, almonds, and pine nuts). They showed that an intake of 15 g/week was inversely related to the risk of MetS in the general Korean population [48].

In the case of pistachio nuts, our results were similar to the values obtained by other authors. Pistachios can be used for dietary supplementation of K, Mg, and Se. Kasliwal et al. conducted a study in which 40 g of pistachios was administered daily to patients with mild changes in lipid metabolism for three months. A reduction in the level of morning fasting glucose and LDL-c concentration was observed, as well as an increase in HDL-c and an improvement in blood vessel stiffness and endothelial function [49]. In healthy people using pistachios in the amount of 42 and 84 g/day for three weeks, LDL-c was reduced [50]. In a study by Hernández-Alonso et al., people with prediabetes ate 57 g of pistachios per day for eight weeks, which resulted in improved insulin sensitivity and decreased expression of several genes (fibrinogen, platelet factor 4, oxidized LDL-c, Interleukin-6 mRNA, and resistin) [51]. A study with two snacks (25 g) was also conducted on diabetic patients. This amount was enough to result in significant changes from the point of view of the disease, i.e., a decrease in glycosylated hemoglobin (HbA1c), blood glucose levels, systolic BP, BMI, and highly sensitive C-reactive protein (hs-CRP) [52]. Another study that also investigated diabetic patients revealed that a 4-week diet with 20% of daily energy from pistachios (from 59 to 128 g) reduced TC, TG, and fructosamine compared to the control diet [53]. The diet with these nuts also showed a beneficial effect in terms of reducing WC, LDL-c, hs-CRP, TNF-alpha, and thiobarbituric acid reactive substances (TBARS), in addition to increasing the concentration of adiponectin in people with MetS [54].

Walnuts, as well as peanuts and hazelnuts, can be labelled as functional products with the capacity to enrich the diet with Mg. In one study, consuming 56 g of walnuts per day for eight weeks improved endothelial function in overweight and visceral obese adults. These results were compared with a control group that consumed an *ad libitum* diet without nuts. The patients did not increase their body weight and even experienced a reduction in their waist circumference (WC) [55]. Ma et al. included walnuts in the diet of subjects accounting 12% of total daily energy consumption for 12 months. Although the patients did not change their eating habits, their plasma lipid profile changed (the concentrations of TC, TG, an LDL-c were reduced) [56]. Spaccarotella et al. reported that in men at risk of prostate cancer, the introduction of 75 g of walnuts per day into the diet improved their biomarkers (decrease in alpha and gamma–tocopherol ratio, increase in gamma–tocopherol and prostate-specific free antigen-PSA) and the vascular health of blood vessels [57]. The effect of walnuts on lipid peroxidation and the function of the endothelium is due to the active substances contained in them, such as α-linolenic acid and alpha-tocopherol [55,56,58,59,60].

Chemometric analyses of nuts described in the literature are primarily related to data on the chromatographic fingerprint [61], interpretation of results obtained by Raman spectroscopy [62] or Fourier transform infrared spectroscopy [63], or a combination of chemometric and metabolomic techniques [64].

This kind of data analysis, especially PCA, can be useful in determining and assessing the relationship between the contents of elements in nuts. In our study, four factors explained 95.0% of the total variation. Kafaoğlu et al. [65] determined the content of 17 elements in nuts an grains, including Ca, Mg, Se, and Zn. In the case of PCA, the authors identified more principal components—six components accounted for a lower percentage (89.1%) of the variance than in our model.

The consumption of nuts in Poland and the relationship with other eating habits and lifestyles were assessed based on data obtained in two multicenter studies: WOBASZ, conducted in 2003–2005; and WOBASZ II, conducted in 2013–2014. Among 12,946 participants, 299 people (2.3%) reported eating nuts. The average consumption in this group was 56.0 ± 52.8 g/day. Nut eaters were distinguished by a higher index of a healthy diet, higher consumption of antioxidants and polyphenols, lower consumption of red meat, lower consumption of processed meats, and more frequent consumption of antiatherosclerotic foods compared to the control group [66].

A strength of our study is that we included almost all nuts available on the market. Secondly, we filled a gap in the literature regarding the content of minerals, especially Se, in macadamia, pecan, and pine nuts. Thirdly, for the first time, an assessment of whether a product can be labelled as a source of a particular mineral was carried out. However, our research is also subject to a limitation. We did not include coconut in the study due to the difficulty in obtaining a representative number of samples resulting from the limited availability of the product.

## 5. Conclusions

A comparison of the most commonly consumed types of nuts showed differences in the content of minerals. In the present study, we confirmed that the studied nuts are valuable products that can be used to supplement the diet with selected minerals and can be labelled as functional products. All analyzed types of nuts can be considered a source of Mg. Moreover, the consumption of almonds is particularly recommended for Ca, K, and Zn; Brazil nuts for Se and Zn; cashews for K, Se, and Zn; hazelnuts for K; macadamia nuts for Se; peanuts, pecans, and pine nuts for Zn; and pistachio nuts for K and Se deficiencies. Including the mentioned nuts in the diet can not only improve the quality of the diet but can also be crucial in the prevention of a multitude of diseases.

## Figures and Tables

**Table 1:** Ca, K, Mg, Se, and Zn contents in various type of nuts.

Type (Sign)	*n*	A.V. ± SD	Med. ^significance relative to signed types in the first column^	Min.–Max.	Q1–Q3
**Ca (mg/kg)**					
Almonds (1)	12	3099 ± 1210	2826 *^7,#3,5,6,8^	948–5407	2428–3836
Brazil nuts (2)	12	2108 ± 670	2166 ^#3,5,6,8^	953–3087	1642–2590
Cashews (3)	12	511 ± 192	501 *^9,‡4,#1,2^	225–851	369–625
Hazelnuts (4)	12	1749 ± 756	1536 ^‡3,6,#5,8^	1163–3923	1323–1828
Macadamia nuts (5)	12	462 ± 185	460 ^#1,2,4,9^	229–853	333–520
Peanuts (6)	12	509 ± 192	582 ^‡4,#1,2,^*^9^	195–775	293–618
Pecans (7)	12	873 ± 240	842 *^1,8^	449–1319	720–1051
Pine nuts (8)	12	144 ± 44	145 *^7,#1,2,4,9,10^	76–202	110–185
Pistachio nuts (9)	12	1413 ± 450	1393 *^3,5,6,#8^	862–2374	1045–1622
Walnuts (10)	12	1121 ± 229	1076 ^#8^	751–1447	969–1348
**K (mg/kg)**					
Almonds (1)	12	10,205 ± 954	10,163 ^#5,‡7^	9170–12,805	9607–10,398
Brazil nuts (2)	12	8124 ± 1951	7990 ^#9^	5509–12,107	6841–8919
Cashews (3)	12	8838 ± 1478	8838 ^‡5,^*^9^	5894–10,837	8298–10,078
Hazelnuts (4)	12	10,545 ± 2438	10,276 ^#5,‡7^	8021–17,506	9393–10,558
Macadamia nuts (5)	12	4630 ± 527	4592 *^6,‡3,#1,4,8,9^	3900–5789	4264–4805
Peanuts (6)	12	8582 ± 601	8584 *^5,‡9^	7024–9483	8459–8931
Pecans (7)	12	6219 ± 2364	5691 ^‡1,4,#8,9^	4445–13,455	5227–6219
Pine nuts (8)	12	10,607 ± 1137	10,607 ^#5,7,^*^10^	8766–12,067	9753–11,830
Pistachio nuts (9)	12	15,627 ± 1806	15,731 *^3,‡6,#2,5,7,10^	10,512–17,968	15,627–16,379
Walnuts (10)	12	7364 ± 756	7364 *^8,#9^	5814–9065	7257–7614
**Mg (mg/kg)**					
Almonds (1)	12	5424 ± 831	5359 *^5,7,#9^	4196–7647	5005–5608
Brazil nuts (2)	12	11,802 ± 3731	10,509 ^#4,5,6,7,9,10^	8221–20,474	9124–13,449
Cashews (3)	12	7086 ± 1891	7281 *^10,#5,7,9^	3699–9394	6037–8847
Hazelnuts (4)	12	3349 ± 1427	2571 ^#2^	2106–6358	2409–4186
Macadamia nuts (5)	12	2092 ± 849	2044 *^1,8,#2,3^	660–3882	1728–2345
Peanuts (6)	12	4324 ± 1274	4049 *^2,9^	2612–6282	3295–5488
Pecans (7)	12	2427 ± 1519	1993 *^1,#2,3^	77–5973	1691–3170
Pine nuts (8)	12	5515 ± 1872	5346 *^5,#9^	2448–8050	3967–7327
Pistachio nuts (9)	12	1514 ± 687	1463 *^6,#1,2,3,8^	214–2884	1088–1908
Walnuts (10)	12	2954 ± 1376	2772 *^3,‡2^	1115–5375	1819–3763
**Se (μg/kg)**					
Almonds (1)	12	36 ± 19	37 *^10,‡5,9,#2,3^	8–63	21–51
Brazil nuts (2)	12	4566 ± 3394	4349 *^6,#1,4,7,8^	126–9390	1666–7290
Cashews (3)	12	669 ± 716	354 ^#1,4^	85–2174	124–875
Hazelnuts (4)	12	50 ± 31	46 *^5,9,#2,3^	14–120	26–69
Macadamia nuts (5)	12	372 ± 314	350 *^4,‡1^	17–1069	96–572
Peanuts (6)	12	161 ± 109	164 *^2^	17–416	80–203
Pecans (7)	12	101 ± 86	80 ^#2^	21–336	44–129
Pine nuts (8)	12	82 ± 43	77 ^#2^	39–205	56–84
Pistachio nuts (9)	12	294 ± 328	165 *^4,‡1^	111–1284	126–282
Walnuts (10)	12	145 ± 36	151 *^1^	88–186	120–178
**Zn (mg/kg)**					
Almonds (1)	12	42 ± 8	42 ^‡5^	32–63	36–45
Brazil nuts (2)	12	61 ± 9	62 ^‡4,10,#5,9^	48–74	54–69
Cashews (3)	12	70 ± 10	67 ^#4,5,9,10^	56–91	64–74
Hazelnuts (4)	12	27 ± 6	27 *^7,‡2,#3,8^	18–34	22–32
Macadamia nuts (5)	12	16 ± 4	16 *^6,‡1,#2,3,7,8^	10–24	14–19
Peanuts (6)	12	39 ± 5	39 *^5^	30–46	35–43
Pecans (7)	12	53 ± 18	50 *^4,#5,9^	31–82	38–71
Pine nuts (8)	12	79 ± 22	72 ^#4,5,9,10^	44–112	70–98
Pistachio nuts (9)	12	22 ± 6	22 ^‡7,#2,3,8^	13–31	19–26
Walnuts (10)	12	28 ± 6	28 ^‡2,#3,8^	20–40	24–31

Abbreviations: A.V.—average value, Ca—calcium, Max.—maximum, Med.—median, Mg—magnesium, Min.—minimum, *n*—number of samples, Q1—quartile 1, Q3—quartile 3, SD—standard deviation, Se—selenium, Zn—zinc. The Kruskal–Wallis test was used to demonstrate statistical significance between medians of the different types of nuts: * *p* < 0.05, ^‡^ *p* < 0.01, ^#^ *p* < 0.001.

**Table 2:** The content of the tested elements in a standard portion of nuts weighing 42 g and percent of RDA, RVI, and INQ for adults.

Type	A.V. ± SD	Med.	Min.–Max.	Q1–Q3	%RDA F	%RDA M	%RVI	INQ F	INQ M
**Ca (mg/kg)**									
Almonds	130.2 ± 50.8	118.7	39.8–227.1	102.0–161.1	13.0		16	1.1	1.3
Brazil nuts	88.5 ± 28.1	91.0	40.0–129.7	69.0–108.8	8.9		11	0.7	0.8
Cashews	21.5 ± 8.1	21.0	9.4–35.7	15.5–26.3	2.1		3	0.2	0.2
Hazelnuts	73.5 ± 31.8	64.5	48.9–164.8	55.6–76.8	7.3		9	0.6	0.7
Macadamia nuts	19.4 ± 7.8	19.3	9.6–35.8	14.0–21.8	1.9		2	0.1	0.2
Peanuts	21.4 ± 8.1	24.5	8.2–32.6	12.3–25.9	2.1		3	0.2	0.2
Pecans	36.7 ± 10.1	35.4	18.9–55.4	30.2–44.1	3.7		5	0.3	0.3
Pine nuts	6.1 ± 1.8	6.1	3.2–8.5	4.6–7.8	0.6		1	0.0	0.1
Pistachio nuts	59.4 ± 18.9	58.5	36.2–99.7	43.9–68.1	5.9		7	0.5	0.6
Walnuts	47.1 ± 9.6	45.2	31.5–60.8	40.7–56.6	4.7		6	0.4	0.4
**K (mg/kg)**									
Almonds	428.6 ± 40.1	426.9	385.1–537.8	403.5–436.7	12.2		21	1.0	1.3
Brazil nuts	341.2 ± 82.0	335.6	231.4–508.5	287.3–374.6	9.7		17	0.7	0.9
Cashews	371.2 ± 62.1	371.2	247.5–455.2	348.5–423.3	10.6		19	1.0	1.2
Hazelnuts	442.9 ± 102.4	431.6	336.9–735.3	394.5–443.4	12.7		22	1.0	1.2
Macadamia nuts	194.5 ± 22.2	192.8	163.8–243.1	179.1–201.8	5.6		10	0.4	0.5
Peanuts	360.4 ± 25.3	360.5	295.0–398.3	355.3–375.1	10.3		18	0.8	1.0
Pecans	261.2 ± 99.3	239.0	186.7–565.1	219.5–261.2	7.5		13	0.7	0.8
Pine nuts	445.5 ± 47.8	445.5	368.2–506.8	409.6–496.9	12.7		22	0.9	1.1
Pistachio nuts	656.3 ± 75.9	660.7	441.5–754.7	656.3–687.9	18.8		33	1.5	1.9
Walnuts	309.3 ± 30.4	309.3	244.2–380.7	305.4–318.0	8.8		15	0.7	0.8
**Mg (mg/kg)**									
Almonds	227.8 ± 34.9	225.1	176.2–321.2	210.2–235.5	73.5	57.0	61	6.1	5.8
Brazil nuts	495.7 ± 156.7	441.4	345.3–859.9	383.2–564.8	159.9	123.9	132	12.2	11.7
Cashews	297.6 ± 79.4	305.8	155.3–394.5	253.5–371.6	96.0	74.4	79	8.7	8.3
Hazelnuts	140.7 ± 59.9	108.0	88.4–267.1	101.2–175.8	45.4	35.2	38	3.4	3.3
Macadamia nuts	87.9 ± 35.7	85.8	27.7–163.0	72.6–98.5	28.3	22.0	23	2.0	1.9
Peanuts	181.6 ± 53.5	170.1	109.7–263.8	138.4–230.5	58.6	45.4	48	4.8	4.6
Pecans	101.9 ± 63.8	83.7	3.2–250.8	71.0–133.1	32.9	25.5	27	2.4	2.3
Pine nuts	231.6 ± 78.6	224.5	102.8–338.1	166.6–307.7	74.7	57.9	62	5.4	5.2
Pistachio nuts	63.6 ± 28.8	61.5	9.0–121.1	45.7–80.1	20.5	15.9	17	1.7	1.6
Walnuts	124.1 ± 57.8	116.4	46.8–225.8	76.4–158.0	40.0	31.0	33	3.0	2.9
**Se (µg/kg)**									
Almonds	1.5 ± 0.8	1.5	0.3–2.7	0.9–2.2	2.8		3	0.3	2.2
Brazil nuts	191.8 ± 142.6	182.6	5.3–394.4	70.0–306.2	348.7		349	26.6	32.9
Cashews	28.1 ± 30.1	14.9	3.6–91.3	5.2–36.7	51.1		51	4.6	5.7
Hazelnuts	2.1 ± 1.3	1.9	0.6–5.0	1.1–2.9	3.8		4	0.3	0.4
Macadamia nuts	15.6 ± 13.2	14.7	0.7–44.9	4.0–24.0	28.4		28	2.0	2.4
Peanuts	6.8 ± 4.6	6.9	0.7–17.4	3.4–8.5	12.3		12	1.0	1.3
Pecans	4.2 ± 3.6	3.4	0.9–14.1	1.8–5.4	7.7		8	0.6	0.7
Pine nuts	3.4 ± 1.8	3.2	1.6–8.6	2.4–3.5	6.2		6	0.5	0.6
Pistachio nuts	12.3 ± 13.8	6.9	4.6–53.9	5.3–11.9	22.4		22	1.8	2.2
Walnuts	6.1 ± 1.5	6.3	3.7–7.8	5.0–7.5	11.0		11	0.8	1.0
**Zn (mg/kg)**									
Almonds	1.8 ± 0.4	1.7	1.3–2.6	1.5–1.9	22.2	16.1	18	1.8	1.7
Brazil nuts	2.6 ± 0.4	2.6	2.0–3.1	2.3–2.9	32.2	23.4	26	2.5	2.2
Cashews	2.9 ± 0.4	2.8	2.3–3.8	2.7–3.1	36.8	26.7	29	3.3	3.0
Hazelnuts	1.1 ± 0.2	1.1	0.8–1.4	0.9–1.3	14.1	10.2	11	1.1	1.0
Macadamia nuts	0.7 ± 0.2	0.7	0.4–1.0	0.6–0.8	8.5	6.2	7	0.6	0.5
Peanuts	1.6 ± 0.2	1.6	1.3–1.9	1.5–1.8	20.2	14.7	16	1.7	1.5
Pecans	2.2 ± 0.8	2.0	1.3–3.5	1.6–3.0	28.0	20.4	22	2.0	1.8
Pine nuts	3.3 ± 0.9	3.0	1.9–4.7	2.9–4.1	41.3	30.0	33	3.0	2.7
Pistachio nuts	0.9 ± 0.2	0.9	0.5–1.3	0.8–1.1	11.5	8.3	9	0.9	0.8
Walnuts	1.2 ± 0.3	1.2	0.8–1.7	1.0–1.3	14.8	10.8	12	1.1	1.0

Abbreviations: A.V.—average value, Ca—calcium, F—female, INQ—index of nutritional quality, M—male, Max.—maximum, Med.—median, Mg—magnesium, Min.—minimum, *n*—number, Q1—quartile 1, Q3—quartile 3, RDA—recommended daily allowance, RVI—reference value intake, SD—standard deviation, Se—selenium, Zn—zinc. RDA: Ca: 1000 mg (for men and women); Mg: 310 mg for women, 400 mg for men; Se: 55 μg (for men and women); Zn: 8 mg for women, 11 mg for men. %RDA as a %AI (adequate intake)—AI for K (3500 mg).

**Table 3:** The amount of nuts (g) that covers 100% of the recommended dietary allowance (RDA).

Type	Ca	Mg		Se	Zn	
		F	M		F	M
Almonds	322.7	57.2	73.7	1524.8	189.4	260.4
Brazil nuts	474.4	26.3	33.9	12.0	130.4	179.3
Cashews	1956.7	43.7	56.4	82.2	114.2	157.1
Hazelnuts	571.6	92.6	119.4	1103.0	298.1	409.9
Macadamia nuts	2162.9	148.2	191.2	147.9	492.3	677.0
Peanuts	1966.3	71.7	92.5	341.0	207.7	285.6
Pecans	1145.2	127.7	164.8	546.2	149.9	206.1
Pine nuts	6940.6	56.2	72.5	673.2	101.8	140.0
Pistachio nuts	707.6	204.8	264.2	187.3	365.8	503.0
Walnuts	891.9	104.9	135.4	380.2	283.2	389.4

Abbreviations: Ca—calcium, F—female, M—male, Mg—magnesium, Se—selenium, Zn—zinc. RDA: Ca: 1000 mg (for men and women); Mg: 310 mg for women, 400 mg for men; Se: 55 μg (for men and women); Zn: 8 mg for women, 11 mg for men.

**Table 4:** Correlations between the contents of the examined elements.

Nuts	Variables	r	*p*-Value
Almonds (*n* = 12)	Mg & K	0.61	<0.05
Brazil nuts (*n* = 12)	Ca & Zn	0.61	<0.05
Cashews (*n* = 12)	Mg & K	0.64	<0.05
Pecans (*n* = 12)	Ca & Zn	0.62	<0.05
Pecans (*n* = 12)	Zn & K	0.62	<0.05
Pine nuts (*n* = 12)	Mg & Zn	0.68	<0.05
Pistachio nuts (*n* = 12)	Mg & Zn	0.59	<0.05
**Total (*n* = 120)**	**Ca & K**	**0.20**	**<0.05**
**Total (*n* = 120)**	**Mg & Zn**	**0.64**	**<0.001**

Abbreviations: Ca—calcium, K—potassium, Mg—magnesium, Se—selenium, r—correlation coefficient, Zn—zinc. Spearman’s rank correlation was used to demonstrate statistically significant relationships between the contents of the examined elements. Bold: the information applies to all samples tested.

**Table 5:** The content of selected minerals in nuts in comparison to literature values.

Type	Med.	L.V.: Med.	A.V. ± SD	L.V.: A.V. ± SD (Min.–Max.)
**Ca (mg/kg)**				
Almonds	2825.8	nd	3099.1 ± 1210.4	1491 ± 41 [67]; 1506 ± 23 [67]; 2096 (154–5686) [68]; 2200 ± 100 [69]; 2690 [70]; 3450 (160–6630) [71]
Brazil nuts	2166.3	1887 [72]	2107.8 ± 670.0	1338 ± 51 [67]; 1461 ± 43 [67]; 1600 [70]; 1703 ± 46.1 [73], 2568 (4–7433) [74]
Cashews	500.7	516 [72]	511.1 ± 192.1	250 ± 30 [69]; 251 ± 18.5 [73]; 450 [70]; 700.2 ± 14.5 [67]; 706.4 ± 24.6 [67]
Hazelnuts	1535.6	1327 [72]	1749.4 ± 756.3	1140 [70]; 1324 ± 43.2 [73]; 1483 ± 6 [67]; 1466 ± 19 [3]
Macadamia nuts	459.8	614 [72]	462.4 ± 185.2	700 [70]; 850 [75]
Peanuts	582.4	726 [72]	508.6 ± 192.0	632.5 ± 7.8 [67]; 639.8 ± 9.3 [67]; 920 [70]
Pecans	841.8	nd	873.3 ± 240.1	700 [70]; 2088 ± 33 [22]
Pine nuts	145.3	286 [72]	144.1 ± 43.9	160 [70]
Pistachio nuts	1392.6	1140 [72]	1413.2 ± 450.4	1000 ± 40 [69]; 1040 [70]; 1279 ± 12 [67]; 1301 ± 29 [67]
Walnuts	1075.9	927 [72]	1121.2 ± 228.6	640 ± 140 [76]; 731 ± 32.2 [73]; 810 ± 80 [76]; 980 [75]; 1062 (866–1435) [77]; 1121 ± 12 [67]; 1146 ± 23 [67]
**K (mg/kg)**				
Almonds	10,163.3	nd	10,204.5 ± 953.6	6500 ± 200 [69]; 6910 (4650–12,350) [71]; 7330 [70]
Brazil nuts	7990.3	nd	8123.6 ± 1951.1	6066 ± 252 [72]; 6590 [70]; 6900 ± 1600 [78];
Cashews	8838.3	nd	8838.3 ± 1477.9	5700 ± 200 [69]; 5650 [70]; 6473 ± 664 [72];
Hazelnuts	10,276.0	nd	10,545.1 ± 2437.7	6800 [70]; 7060 ± 313 [72];
Macadamia nuts	4591.7	nd	4630.3 ± 527.4	3545 ± 188 [72]; 3630 [70]
Peanuts	8583.8	nd	8581.5 ± 601.2	7050 [70]; 7406 ± 241 [72];
Pecans	5690.5	nd	6218.7 ± 2364.3	4100 [70]
Pine nuts	10,606.5	nd	10,607.0 ± 1137.0	5970 [70]; 7684 ± 1601 [72];
Pistachio nuts	15,730.5	nd	15,626.5 ± 1805.9	9770 [70]; 10,100 ± 200 [69]
Walnuts	7363.6	nd	7364.1 ± 756.4	2771 (2006–3221) [77]; 3750 ± 220 [76]; 4410 [70]; 6139 ± 838 [72]
**Mg (mg/kg)**				
Almonds	5358.8	nd	5424.3 ± 831.2	2360 (1590–3340) [71]; 2477 ± 41 [67]; 2500 ± 40 [69]; 2554 ± 27 [67]; 2650 (197–5286) [68]; 5424 ± 52 [22]
Brazil nuts	10,509.2	5307 [72]	11,801.8 ± 3731.3	2212 ± 64 [73]; 2869 ± 19 [67]; 2957 ± 23 [67]; 3935 (4–9679) [74]; 9679 ± 69 [22]
Cashews	7281.0	1755 [72]	7086.3 ± 1890.5	1957 ± 37.4 [73]; 2000 ± 50 [69]; 2297 ± 26 [67]; 2444 ± 33 [67]
Hazelnuts	2571.1	807 [72]	3349.4 ± 1426.5	1400 ± 53.3 [73]; 1497 ± 23 [67]; 1524 ± 12 [67]; 1630
Macadamia nuts	2043.6	532 [72]	2092.4 ± 849.1	1180; 1300 [75]; 4887 ± 24 [22]
Peanuts	4049.2	1284 [72]	4323.8 ± 1273.8	1680; 2036 ± 23 [67]; 2079 ± 37 [67]
Pecans	1993.1	nd	2427.1 ± 1519.3	1210; 4197 ± 61 [22]
Pine nuts	5345.8	1659 [72]	5514.6 ± 1871.5	2510
Pistachio nuts	1463.3	631 [72]	1513.9 ± 686.8	1000 ± 30 [69]; 1757 ± 29 [67]; 1893 ± 19 [67]
Walnuts	2772.0	874 [72]	2954.0 ± 1375.8	570 ± 120 [76]; 720 ± 150 [76]; 1401 ± 41.7 [73]; 1426 (875–1824) [77]; 1557 ± 31 [3]; 1580 [75]; 1592 ± 7 [67]
**Se (µg/kg)**				
Almonds	36.8	nd	36.1 ± 18.8	570 ± 180 [79]; 765.1 ± 86.3 [67]; 796.7 ± 91.3 [67]
Brazil nuts	4348.7	nd	4566.2 ± 3394.0	763.5 ± 66.8 [67]; 806.1 ± 53.7 [67]; (2070–68,150) [80]
Cashews	353.9	nd	668.9 ± 716.1	922.0 ± 88.6 [67]; 937.2 ± 62.1 [67]
Hazelnuts	45.8	nd	49.9 ± 31.0	723.3 ± 52.1 [67]; 742.6 ± 90.1 [67]
Macadamia nuts	349.9	nd	371.9 ± 313.9	nd
Peanuts	163.9	nd	161.3 ± 109.1	712.2 ± 73.2 [67]; 738.7 ± 89.2 [67]
Pecans	80.3	nd	100.7 ± 85.6	nd
Pine nuts	76.5	nd	81.7 ± 42.7	nd
Pistachio nuts	164.9	nd	293.7 ± 328.4	160 ± 160 [79]; 658.3 ± 85.2 [67]; 752.4 ± 73.1 [67]
Walnuts	150.5	nd	144.7 ± 35.7	194 (77–301) [77]; 892.3 ± 73.1 [67]; 979.1 ± 92.2 [67]
**Zn (mg/kg)**				
Almonds	42.0	nd	42.2 ± 8.3	24 ± 4.0 [79]; 34.1 ± 1.0 [69]; 45.38 ± 0.08 [67]; 46.71 ± 0.12 [67]; 50 ± 0.09 [22]
Brazil nuts	61.6	61.6 [72]	61.3 ± 8.6	24 ± 1.1 [73]; 35.22 ± 0.35 [67]; 36.71 ± 0.16 [67]; 47 (6–110) [74]
Cashews	67.2	76.2 [72]	70.0 ± 10.2	30 ± 2.4 [73]; 41 ± 12 [79]; 42.1 ± 0.4 [69]; 58.29 ± 0.44 [67]; 59.33 ± 0.72 [67]
Hazelnuts	27.1	49.0 [72]	26.8 ± 5.5	15 ± 0.2 [73]; 15 ± 0.82 [79]; 34.43 ± 0.10 [67]; 36.01 ± 0.61 [67]
Macadamia nuts	15.7	37.4 [72]	16.2 ± 4.1	8.6 ± 1.5 [79]; 13 [75]; 39 ± 0.7 [22]
Peanuts	38.9	65.2 [72]	38.5 ± 4.9	41.02 ± 0.67 [67]; 43.63 ± 0.22 [67]
Pecans	49.6	nd	53.4 ± 18.1	138 ± 0.40 [22]
Pine nuts	72.4	79.7 [72]	78.6 ± 22.0	38 ± 8.6 [79]
Pistachio nuts	21.5	46.2 [72]	21.9 ± 5.5	15 ± 3.3 [79]; 23.8 ± 0.9 [69]; 33.99 ± 0.67 [67]; 34.61 ± 0.12 [67]
Walnuts	28.4	51.0 [72]	28.3 ± 6.0	18 ± 0.3 [73]; 20 ± 1.1 [79]; 24.0 (20.3–32.8) [77]; 24.90 ± 3.34 [76]; 26.70 ± 3.89 [81]; 30.9 [75]; 31.46 ± 3.44 [76]; 34.91 ± 0.11 [67]; 36.27 ± 0.76 [67]

Abbreviations: A.V.—average value, Ca—calcium, K—potassium, L.V.—literature value, Max.—maximum, Med.—median, Mg—magnesium, Min.—minimum, *n*—number, nd—no data, SD—standard deviation, Se—selenium, Zn—zinc.

## Data Availability

The data supporting the reported results are available from the corresponding author upon reasonable request.
